# Human Immunodeficiency Virus-Associated Exosomes Promote Kaposi’s Sarcoma-Associated Herpesvirus Infection via the Epidermal Growth Factor Receptor

**DOI:** 10.1128/JVI.01782-19

**Published:** 2020-04-16

**Authors:** Lechuang Chen, Zhimin Feng, Guoxiang Yuan, Corey C. Emerson, Phoebe L. Stewart, Fengchun Ye, Ge Jin

**Affiliations:** a Department of Biological Sciences, Case Western Reserve University School of Dental Medicine, Cleveland, Ohio, USA; b Department of Pharmacology, Case Western Reserve University School of Medicine, Cleveland, Ohio, USA; c Cleveland Center for Membrane and Structural Biology, Case Western Reserve University School of Medicine, Cleveland, Ohio, USA; d Department of Molecular Biology and Microbiology, Case Western Reserve University School of Medicine, Cleveland, Ohio, USA; e Center for AIDS Research, Case Western Reserve University and University Hospitals Cleveland Medical Center, Cleveland, Ohio, USA; f Case Comprehensive Cancer Center, Case Western Reserve University, Cleveland, Ohio, USA; University of Southern California

**Keywords:** EGFR, HIV, HIV TAR RNA, KSHV, cetuximab, exosomes, extracellular vesicles, infection, oral, saliva

## Abstract

Kaposi’s sarcoma-associated herpesvirus (KSHV) is the causal agent for Kaposi’s sarcoma (KS), the most common malignancy in people living with human immunodeficiency virus (HIV)/AIDS. The oral cavity is a major route for KSHV infection and transmission. However, how KSHV breaches the oral epithelial barrier for spreading to the body is not clear. Here, we show that exosomes purified from either the saliva of HIV-positive individuals or the culture supernatants of HIV-1-infected T-cell lines promote KSHV infectivity in immortalized and primary human oral epithelial cells. HIV-associated saliva exosomes contain the HIV *trans*-activation response element (TAR), Tat, and Nef RNAs but do not express Tat and Nef proteins. The TAR RNA in HIV-associated exosomes contributes to enhancing KSHV infectivity through the epidermal growth factor receptor (EGFR). An inhibitory aptamer against TAR RNA reduces KSHV infection facilitated by the synthetic TAR RNA in oral epithelial cells. Cetuximab, a monoclonal neutralizing antibody against EGFR, blocks HIV-associated exosome-enhanced KSHV infection. Our findings reveal that saliva containing HIV-associated exosomes is a risk factor for the enhancement of KSHV infection and that the inhibition of EGFR serves as a novel strategy for preventing KSHV infection and transmission in the oral cavity.

**IMPORTANCE** Kaposi’s sarcoma-associated herpesvirus (KSHV) is the causal agent for Kaposi’s sarcoma (KS), the most common malignancy in HIV/AIDS patients. Oral transmission through saliva is considered the most common route for spreading the virus among HIV/AIDS patients. However, the role of HIV-specific components in the cotransfection of KSHV is unclear. We demonstrate that exosomes purified from the saliva of HIV-positive patients and secreted by HIV-infected T-cell lines promote KSHV infectivity in immortalized and primary oral epithelial cells. HIV-associated exosomes promote KSHV infection, which depends on HIV *trans*-activation response element (TAR) RNA and EGFR of oral epithelial cells, which can be targeted for reducing KSHV infection. These results reveal that HIV-associated exosomes are a risk factor for KSHV infection in the HIV-infected population.

## Abstract (precis)

Kaposi’s sarcoma-associated herpesvirus (KSHV) is the causal agent for Kaposi’s sarcoma (KS), the most common malignancy in HIV/AIDS patients. Oral transmission through saliva is considered the most common route for spreading the virus among HIV/AIDS patients. However, the role of HIV-specific components in the cotransfection of KSHV is unclear. We demonstrate that exosomes purified from the saliva of HIV-positive patients and secreted by HIV-infected T-cell lines promote KSHV infectivity in immortalized and primary oral epithelial cells. HIV-associated exosomes promote KSHV infection, which depends on HIV *trans*-activation response element (TAR) RNA and EGFR of oral epithelial cells, which can be targeted for reducing KSHV infection. These results reveal that HIV-associated exosomes are a risk factor for KSHV infection in the HIV-infected population.

## INTRODUCTION

Kaposi’s sarcoma (KS), the most common malignancy in patients infected with human immunodeficiency virus (HIV), is etiologically associated with infection by Kaposi’s sarcoma-associated herpesvirus (KSHV), also known as human herpesvirus 8 (HHV-8) (1). This oncogenic gammaherpesvirus is also linked with primary effusion lymphoma (PEL), multicentric Castleman’s disease (MCD), and KSHV inflammatory cytokine syndrome (KICS) in aging people and immunocompromised adults (2). Oral transmission of KSHV through saliva, in particular, is believed to be the most common route for spreading the virus among men who have sex with men and by mother-to-child transmission (3, 4). The oral mucosa is the first target of KSHV infection, once the virus is in the oral cavity (5–9). Although the KS incidence has dramatically decreased in developed countries in the era of antiretroviral therapy (ART), KS remains the most frequent tumor in the HIV-infected population worldwide (10–12). The oral milieu of HIV-infected patients has long been deduced to favor KSHV infection; however, the role of HIV in KSHV infection and transmission is largely unknown.

Most types of cells can release lipid membrane-enclosed vesicles, generally called extracellular vesicles (EVs), into the extracellular space and body fluids. Saliva and other body fluids, including the blood and breast milk, contain a variety of EVs (13–16). EVs are highly heterogeneous and dynamic and can be generally grouped into exosomes (17–20), macrovesicles (21), and apoptotic bodies, based on biogenesis and the origin of vesicles (22). Exosomes are generated as intraluminal vesicles that bud away from the cytoplasm into an intermediate endocytic compartment termed the multivesicular body (MVB) and then shed from cells upon fusion of the MVB with the plasma membrane (17, 19, 20). Exosomes contain molecular cargos of their cells of origin, including proteins and RNAs (20). Although commonly used exosome purification protocols often coisolate different types of EVs, the differential ultracentrifugation method isolates EVs that contain tetraspanins (CD63, CD81, and CD9) and other endosome marker-enriched vesicles, which are characteristics of exosomes (20, 23). Exosomes derived from the culture supernatants of latently HIV-1-infected T-cell clones do not contain HIV-1 particles, although these vesicles do contain viral proteins, such as Gag and the precursor form of the Env protein (p160) (24). The HIV proviral genome can produce *trans*-activation response element (TAR) RNA, which folds in the nascent transcript and facilitates the binding of the viral transcriptional *trans*-activator (Tat) protein to enhance transcription initiation and the elongation of HIV (25). Exosomes isolated from HIV-1-infected cells or HIV-infected (HIV^+^) patients’ sera also contain TAR RNA in vast excess of the amount of total viral RNA (24, 26). TAR RNA-bearing exosomes can induce proinflammatory cytokines in primary macrophages (27) and stimulate proliferation, migration, and invasion of head and neck and lung cancer cells in an epidermal growth factor receptor (EGFR)-dependent manner (26). Exosomes in the body fluids of HIV/AIDS patients may mediate HIV-1 RNA and protein trafficking and affect HIV pathogenesis (28, 29). However, the role of salivary HIV-associated exosomes in the infection of KSHV has not been explored (28).

Here, we report that exosomes purified from either the saliva of people living with HIV or the culture medium of latently HIV-infected T-cell lines enhance KSHV infectivity in human oral epithelial cells (HOECs) cultured in both monolayer and 3-dimensional (3-D) formats. Exosomes from T cells latently infected with an HIV-1 isolate that contains a dysfunctional mutant HIV Tat and the deletion of the Nef gene can still stimulate KSHV infection. Although HIV-associated exosomes lack the viral proteins that are involved in cellular processes, they contain the HIV TAR RNA in an amount in excess of that of other HIV RNAs. We demonstrate that both TAR RNA alone and TAR RNA-bearing exosomes enhance KSHV infectivity in oral epithelial cells, indicating the importance of HIV TAR RNA in promoting KSHV infection. TAR RNA-enhanced KSHV infection is reduced by an aptamer against the TAR RNA. HIV-associated exosome-enhanced KSHV infection is blocked by a monoclonal antibody against EGFR. Epidermal growth factor (EGF) treatment also increases KSHV infectivity in oral epithelial cells. Our findings reveal that HIV-associated saliva exosomes are a risk factor for the enhancement of KSHV infection and that inhibition of EGFR serves as a novel strategy for controlling KSHV infection and transmission in the oral cavity.

## RESULTS

### HIV-associated exosomes enhance KSHV infectivity in oral epithelial cells.

**FIG 1:**
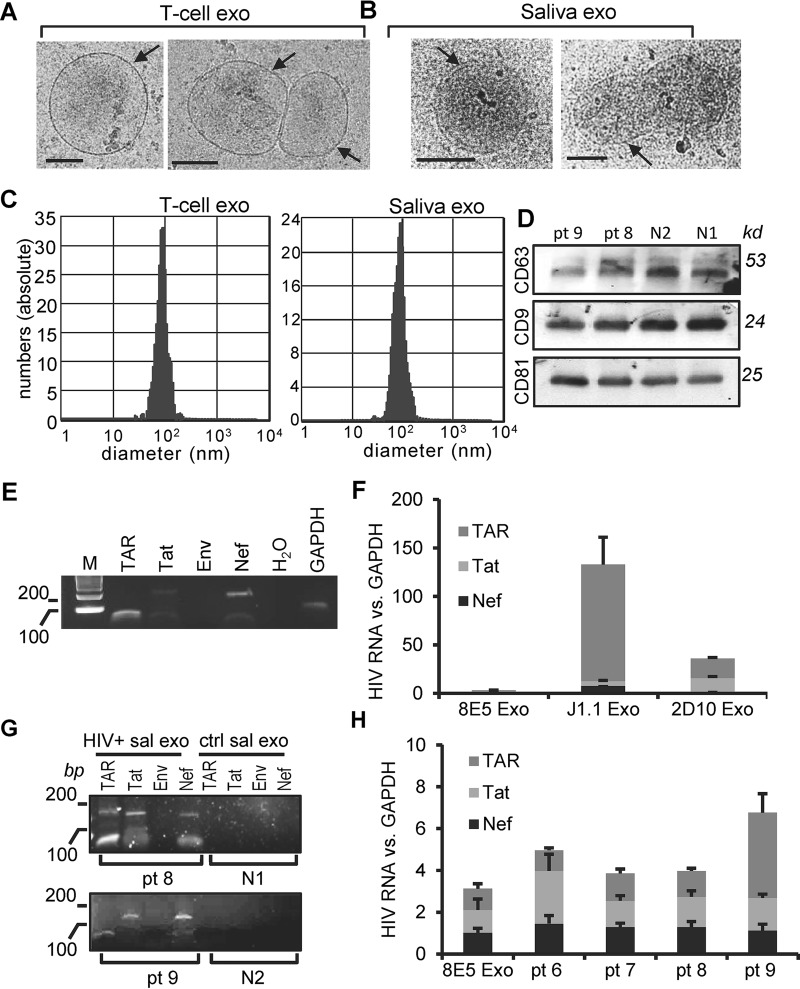
Characterization of HIV-associated saliva exosomes. (A and B) Cryo-EM images of exosomes (exo) from Jurkat T cells (A) and the saliva of healthy donors (B). Arrows, exosome membrane. Bars, 100 nm. (C) Size distribution of exosomes from the culture supernatants of Jurkat T cells (T-cell exo) and the saliva of healthy donors (Saliva exo) determined with the ZetaView nanoparticle tracking analyzer. (D) Immunoblot images of total proteins extracted from saliva exosomes from HIV^+^ (pt 8 and pt 9) and healthy (N1 and N2) donors. *kd*, kilodaltons. (E) RT-PCR gel images of the HIV TAR (60 bp), Tat (192 bp), Env (168 bp), and Nef (175 bp) PCR products in exosomes isolated from the culture supernatants of J1.1 cells. Lane M, molecular size markers (with the numbers to the left indicating molecular sizes [in base pairs]). (F) Quantitative RT-PCR of TAR, Tat, and Nef RNAs on total RNA extracted from exosomes secreted from HIV^+^ 8E5/LAV (8E5), J1.1, and 2D10 T cells. The levels of HIV RNAs of exosomes from J1.1 and 2D10 cells were compared to those of exosomes from 8E5/LAV cells containing a single copy of the HIV proviral genome. (G) RT-PCR gel images of HIV TAR, Tat, Env, and Nef amplimers in saliva exosomes (sal exo) purified from HIV^+^ (pt 8 and pt 9) and healthy (N1 and N2) donors. Note a nonspecific, ∼170-bp PCR band in the TAR lane for pt 8. (H) Quantitative RT-PCR of TAR, Tat, and Nef RNAs in exosomes purified from the saliva of HIV^+^ donors (pt 6 to pt 9). Exosomal RNA from HIV^+^ 8E5/LAV cells (8E5 Exo) was used as a control. The levels of each HIV RNA over those of GAPDH (glyceraldehyde-3-phosphate dehydrogenase) RNA in exosomes were quantified.

We purified exosomes from the whole saliva of healthy donors and persons living with HIV (PLWH) using the differential ultracentrifugation protocol (26). T-cell exosomes showed the typical donut shape in negative-stain transmission electron microscopy (TEM) images, with their sizes ranging from 25 to 250 nm in diameter, as we previously reported (26). Cryo-electron microscopy (cryo-EM) images revealed that exosomes from Jurkat T cells and the saliva of healthy donors are spheroid or irregularly shaped (Fig. 1A and B, arrows). While both T-cell and saliva exosomes have a lipid bilayer/membrane, saliva exosomes have an electron-dense structure (Fig. 1B). The cryo-EM images also presented numerous surface-exposed membrane proteins. We determined the size distribution of T-cell and saliva exosomes using a ZetaView nanoparticle tracking analyzer. The results of nanoparticle tracking analysis (NTA) indicated that over 90% of the nanoparticles were in the size range of 50 to 80 nm in diameter (Fig. 1C; left, T-cell exosomes; right, saliva exosomes). The concentrations of saliva exosomes from 3 healthy donors ranged from 6.3 × 10^10^ to 2.3 × 10^11^/ml, while those from 3 independently prepared culture supernatants of J1.1 cells ranged from 4 × 10^9^ to 8 × 10^9^/ml, based on NTA. We have used the acetylcholinesterase (AChE) activities of T-cell extracellular vesicles to quantify exosomes (26). We found that the concentration of T-cell exosomes quantified using the AChE assay was the same as that determined by the NTA measurement. The total protein levels of saliva exosomes were correlated with the exosome concentrations determined with the ZetaView nanoparticle tracking analyzer. In this study, we treated cells with T-cell exosomes at a final concentration of 4 × 10^9^ exosomes/ml or saliva exosomes at 100 μg/ml of exosomal proteins, which was equivalent to 4 × 10^9^ to 5 × 10^9^/ml of exosomes, as quantified with the ZetaView nanoparticle tracking analyzer. T-cell EVs express proteins of the tetraspanin family (CD9, CD63, and CD81), which are markers of exosomes (26). To determine if saliva EVs also contained these markers, we performed an immunoblot assay with the total proteins of saliva exosomes from HIV-positive and HIV-negative (HIV^−^) donors and found that saliva EVs contained the CD63, CD9, and CD81 proteins (Fig. 1D). These results indicate that our preparations of saliva EVs contain exosomes. As reported before, HIV RNAs were readily detected using reverse transcription-PCR (RT-PCR) in exosomes from the culture supernatants of HIV-1-infected J1.1 T cells (Fig. 1E) (26). Quantitative RT-PCR on total RNA extracted from exosomes secreted from HIV^+^ 8E5/LAV, J1.1, and 2D10 T cells (26) demonstrated that J1.1 and 2D10 cell exosomes contained significantly higher levels of TAR RNA than Tat and Nef RNA (Fig. 1F). To determine whether HIV^+^ saliva exosomes contained HIV-specific cargo components, we performed RT-PCR on total exosomal RNA. We found that HIV^+^ saliva exosomes contained only HIV TAR, Tat, and Nef RNA and not Env RNA (Fig. 1G). Quantitative RT-PCR of the total RNA extracted from HIV^+^ saliva exosomes revealed that the expression levels of HIV TAR, Tat, and Nef RNA were similar to or higher than those in exosomes from cells of the HIV^+^ 8E5/LAV T-cell line, which is an HIV-infected CD4^+^ human CEM T-cell line that contains a single copy of integrated HIV proviral genome (30) (Fig. 1H). Our findings indicate that the saliva of HIV^+^ patients, despite ART, contains HIV-associated exosomes.

### HIV^+^ saliva exosomes promote KSHV infection in immortalized and primary human oral epithelial cells.

**FIG 2:**
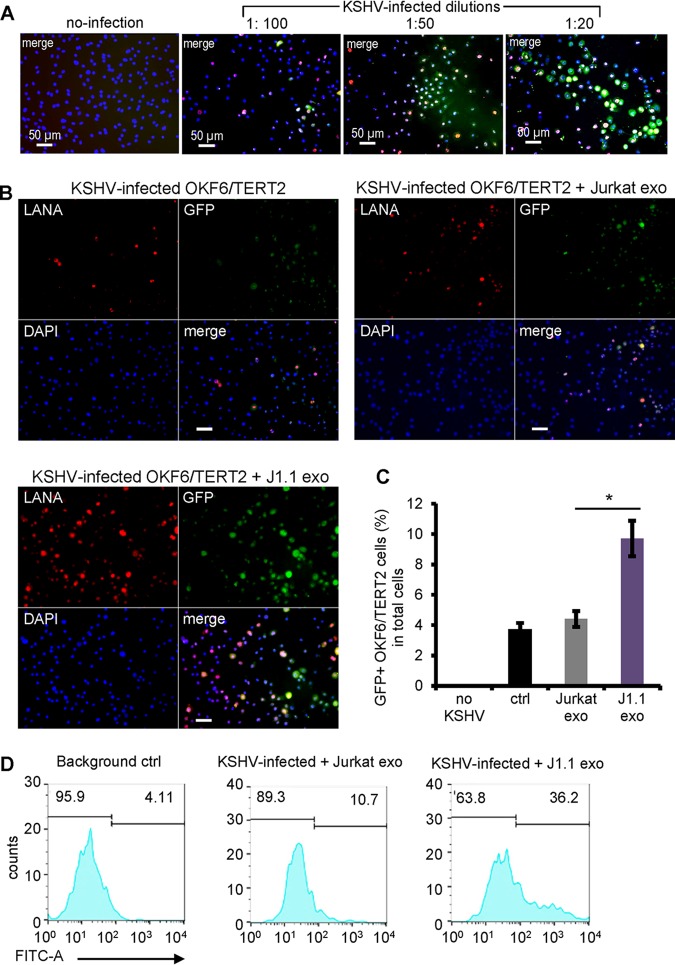
HIV^+^ exosomes promote KSHV infection in oral epithelial cells. (A) Serial dilution of KSHV infection in OKF6/TERT2 cells. A freshly prepared KSHV stock solution was diluted to 1:100, 1:50, and 1:20 with the culture medium for infection in OKF6/TERT2 cells. At 20 h after infection, cells were stained using antibodies to LANA and GFP. Immunofluorescent images were taken using a fluorescence microscope. (B) OKF6/TERT2 cells were treated with exosomes from HIV^+^ J1.1 (J1.1 exo) or Jurkat (Jurkat exo) cells at 4 × 10^9^ exosomes/ml for 10 min, followed by KSHV infection for 20 h. Cells were fixed for immunofluorescent staining using antibodies to LANA (red) and GFP (green). Bars, 25 μm. (C) Flow cytometry of GFP^+^ cells after KSHV infection in the presence of exosomes from J1.1 and Jurkat cells, respectively. The control (ctrl) consisted of KSHV alone. Data represent the results of one independent experiment (*n* = 4) out of three biological repeats. *, *P* < 0.05, F test. (D) Flow cytometry histogram of the results presented in panel C. FITC-A, fluorescein isothiocyanate area.

We treated iSLK-BAC16 cells (31) with sodium butyrate and doxycycline to produce infectious KSHV virions. The recombinant KSHV contains a human elongation factor 1 alpha (EF1α)-driven green fluorescent protein (GFP) expression cassette; thus, the KSHV-infected cells are GFP positive (GFP^+^) (31). KSHV can effectively infect cultured oral epithelial cells, as shown by expression of the KSHV latency-associated nuclear antigen (LANA) (32, 33). To optimize the condition for KSHV infection, we infected cells of the immortalized oral epithelial cell line OKF6/TERT2 (34) with the KSHV virion stock at 1:100, 1:50, and 1:20 dilutions for 20 h, followed by immunofluorescent staining of KSHV LANA (35) and GFP (Fig. 2A). We estimated that about 25% of the oral epithelial cells were infected with the 1:100 dilution of the KSHV stock solution. We tested each batch of the KSHV preparations and used the dilution of KSHV stock to ensure that 15% to 25% of the cells were infected throughout all infection assays.

**FIG 3:**
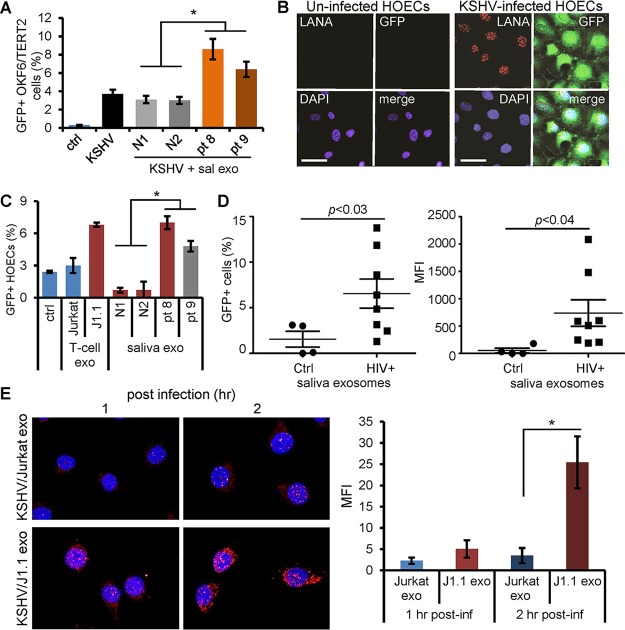
HIV-associated saliva exosomes enhance KSHV infection in oral epithelial cells. (A) OKF6/TERT2 cells were treated with HIV^+^ (pt 8 and pt 9) and HIV^−^ (N1 and N2) saliva exosomes (100 μg/ml of exosome protein) for 10 min and then infected with KSHV for 20 h. Infection was quantified by GFP flow cytometry. *, *P* < 0.05. (B) Human oral epithelial cells (HOECs) were infected with KSHV virion solution without dilution to test the infectivity of KSHV in primary HOECs. LANA (red) and GFP (green) expression was detected using immunofluorescent staining. Nuclei stained blue (DAPI). Bars, 50 μm. Representative images are shown. (C) HOECs were treated with saliva exosomes (100 μg exosomal protein/ml) from healthy (N1 and N2) and HIV^+^ (pt 8 and pt 9) donors, followed by KSHV infection. KSHV-infected GFP^+^ HOECs were quantified using flow cytometry. Data represent the mean ± SD. *, *P* < 0.05. (D) HOECs were treated with saliva exosomes (100 μg/ml of exosome protein) purified from healthy (*n* = 4) and HIV-infected (*n* = 8) donors, followed by KSHV infection. KSHV-infected GFP^+^ HOECs were determined by flow cytometry. (Right) Mean fluorescence intensity (MFI) plot of the data in the left panel. *P* values were determined by one-way ANOVA. (E) (Left) OKF6/TERT2 cells were infected with KSHV in the presence of Jurkat cell exosomes (Jurkat exo) or HIV^+^ J1.1 cell exosomes (J1.1 exo) for 1 and 2 h, followed by immunofluorescent staining of ORF65 (red). Representative images are shown. (Right) MFI of ORF65 staining in OKF6/TERT2 cells. Data represent those from one independent experiment (*n* = 4). *, *P* < 0.05, F test. post-inf, postinfection.

To determine whether HIV^+^ exosomes were able to affect KSHV infection in oral epithelial cells, we incubated OKF6/TERT2 cells with KSHV virions in the presence of exosomes isolated from the culture supernatants of latently HIV-1-infected (HIV^+^) J1.1 T cells or HIV-negative (HIV^−^) Jurkat cells (26, 36). HIV^+^ exosomes from J1.1 cells significantly enhanced KSHV infection compared to those from the control Jurkat T cells, as shown by immunofluorescence microscopy of LANA and GFP (Fig. 2B). The enhancement of KSHV infection by HIV^+^ exosomes was confirmed by flow cytometry on GFP of infected cells (Fig. 2C and D). Our results suggest that HIV^+^ exosomes enhance KSHV infection in oral epithelial cells. KSHV infects the oral cavity and oropharynx, and the infection is more prevalent in HIV-positive persons than in the general population (9, 37). We postulated that HIV-associated saliva exosomes might be responsible for the higher incidence of KSHV infection in persons living with HIV/AIDS. To test this hypothesis, we purified saliva exosomes from HIV-infected donors with ongoing ART and CD4 T-cell counts over 400 per ml and those from healthy individuals, using the differential ultracentrifugation protocol (26). Saliva exosomes from HIV-positive subjects (Fig. 3A, patient 8 [pt 8] and pt 9) significantly enhanced KSHV infection in OKF6/TERT2 cells compared to those from healthy individuals (Fig. 3A, N1 and N2), as measured by GFP flow cytometry. To determine whether KSHV was able to infect primary human oral epithelial cells (HOECs), we treated the cells with the KSHV virion stock solution. We found that KSHV was able to infect HOECs and that the infected cells expressed both KSHV LANA and GFP (Fig. 3B). To test whether HIV^+^ exosomes were able to enhance KSHV infection in primary oral epithelial cells, HOECs were treated with exosomes from HIV^+^ J1.1 and HIV^−^ Jurkat cells as well as those purified from the saliva of HIV^+^ and HIV^−^ donors. Exosomes isolated from HIV^+^ T cells or those from the saliva of HIV-positive donors significantly stimulated KSHV infection in HOECs compared to exosomes from control T cells or the saliva of healthy donors (Fig. 3C). To verify the stimulatory effect of HIV-associated saliva exosomes on KSHV infection, we collected unstimulated whole-saliva samples from HIV-positive donors (*n* = 8) who were under ART with CD4 T-cell counts of over 400 per ml and a viral load of 50 to 6,000 or from healthy individuals for exosome purification. HOECs were treated with saliva exosomes for 10 min and then tested by the KSHV infection assay. KSHV infection was considerably enhanced by HIV^+^ saliva exosomes compared to HIV^−^ saliva exosomes in primary HOECs (Fig. 3D, left, percentage of infected cells, and right, mean fluorescence intensity [MFI]). We reasoned that the elevated KSHV infectivity by HIV^+^ exosomes could result from increased viral entry or enhanced viral gene expression and replication, or both. We therefore examined the effect of HIV^+^ exosomes on viral entry by measuring KSHV virions with a monoclonal antibody against the small capsid protein (ORF65) (38) in OKF6/TERT2 cells at 1 and 2 h after KSHV infection in the presence of J1.1 or Jurkat cell exosomes. HIV^+^ J1.1 T-cell exosome treatment significantly increased ORF65-positive KSHV virions in OKF6/TERT2 cells at both 1 and 2 h postinfection compared with Jurkat cell exosome treatment (Fig. 3E, right, MFI of infected cells). Collectively, these results demonstrate that HIV^+^ saliva exosomes indeed promote viral entry during the early infection time in oral epithelial cells to enhance *de novo* KSHV infection.

### HIV^+^ saliva exosomes promote KSHV infection and transmission in organotypic culture models of oral mucosa.

**FIG 4:**
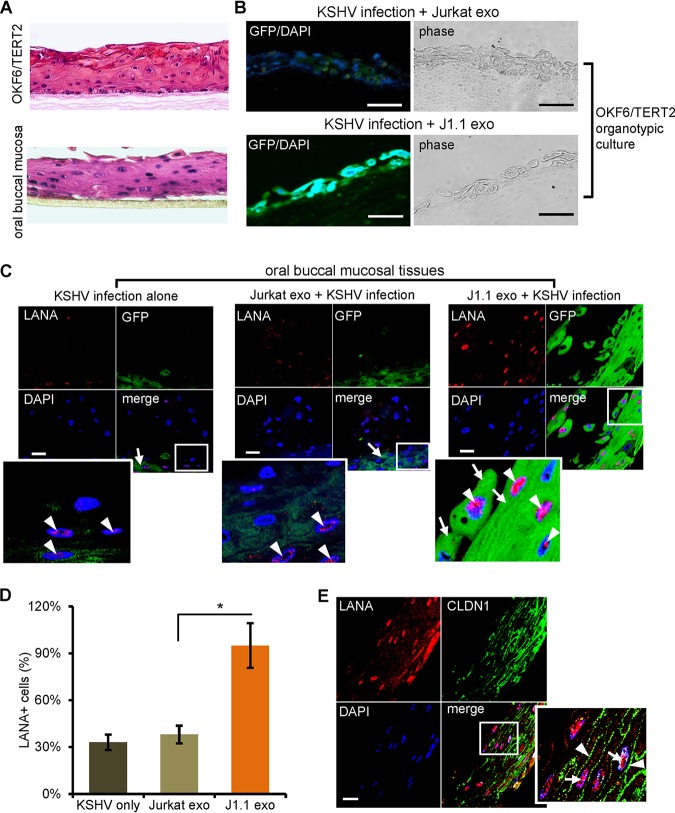
HIV^+^ exosomes increase KSHV infection in oral mucosal tissue cultures. (A) Hematoxylin and eosin (H&E) staining of the organotypic culture of OKF6/TERT2 cells (top) and the oral buccal mucosal tissue consisting of primary human oral epithelial cells (MatTek Inc.) (bottom). Representative images are shown. (B) HIV^+^ J1.1 T-cell exosomes (J1.1 exo) increased KSHV infection in OKF6/TERT cells grown in the organotypic culture model. GFP^+^ cells represent KSHV-infected cells. Representative images are shown. (C) Cultured oral buccal mucosal tissues were treated with HIV^+^ (J1.1 exo) and HIV^−^ (Jurkat exo) exosomes, followed by KSHV infection. Tissue sections were stained with antibodies to LANA (red) and GFP (green). Nuclei stained blue (DAPI). Arrows, GFP; arrowheads, LANA. Bars, 25 μm. Representative images are shown. The lower left image represents the zoomed-in box of each merge photo to detail cellular expression of GFP and LANA. (D) Quantification of LANA-positive cells versus total cells in 3 to 5 sections of each paraffin block shown in panel C. Data are represented as the mean ± SD. *, *P* < 0.05. (E) The oral mucosal tissue (MatTek Inc.) treated with J1.1 cell exosomes and infected with KSHV was stained for LANA (red) and the tight junction protein claudin 1 (CLDN1; green). Arrows in the enlarged inset of the boxed area, LANA; arrowheads in enlarged inset, claudin 1. Bar, 25 μm.

The oral mucosa is the first target of KSHV infection, once the virus is in the oral cavity (5, 6, 8, 9). To evaluate the role of HIV-associated exosomes in KSHV infection through the oral mucosa, we created a 3-dimensional (3-D) organotypic culture by using OKF6/TERT2 cells, as described previously (39) (Fig. 4A, top), followed by KSHV infection. The paraffin blocks were then sectioned and stained for GFP. We found that HIV^+^ J1.1 cell exosomes, but not HIV^−^ Jurkat cell exosomes, promoted KSHV infection in cells of the organotypic culture (Fig. 4B). Further, we infected 3-D cultured oral buccal mucosal tissues consisting of primary human oral epithelial cells (MatTek Co., Ashland, MA), which display the anatomic structures of stratified mucosa (Fig. 4A, bottom). Treatment with exosomes from HIV^+^ J1.1 T cells increased the numbers of cells expressing the LANA protein (Fig. 4C, arrowheads) and GFP in the mucosal tissue compared with the numbers obtained by treatment with HIV^−^ exosomes from Jurkat cells (Fig. 4C, arrows). To quantify KSHV infection in the oral mucosal tissue, we calculated the number of LANA-positive cells in 3 to 5 consecutive sections of each mucosal tissue block. Treatment with HIV^+^ J1.1 T-cell exosomes significantly increased the number of LANA-expressing cells compared to the number obtained by treatment with HIV^−^ Jurkat T-cell exosomes (Fig. 4D). To evaluate if KSHV virions were able to infect through the fully established oral epithelium, we stained the J1.1 cell exosome-treated, KSHV-infected 3-D oral mucosal culture for the protein claudin 1 (CLDN1) (40) and the KSHV LANA. Nuclear expression of LANA expression (Fig. 4E, arrowheads) was seen in mucosal epithelial cells expressing CLDN1 (Fig. 4E, arrows), a tight junction protein that is involved in the maintenance of epithelial integrity and the function of differentiated epithelia (40). Collectively, these findings suggest that HIV^+^ exosomes promote KSHV transmission through the oral mucosa.

### HIV TAR RNA is the key element for enhancement of KSHV infection.

**FIG 5:**
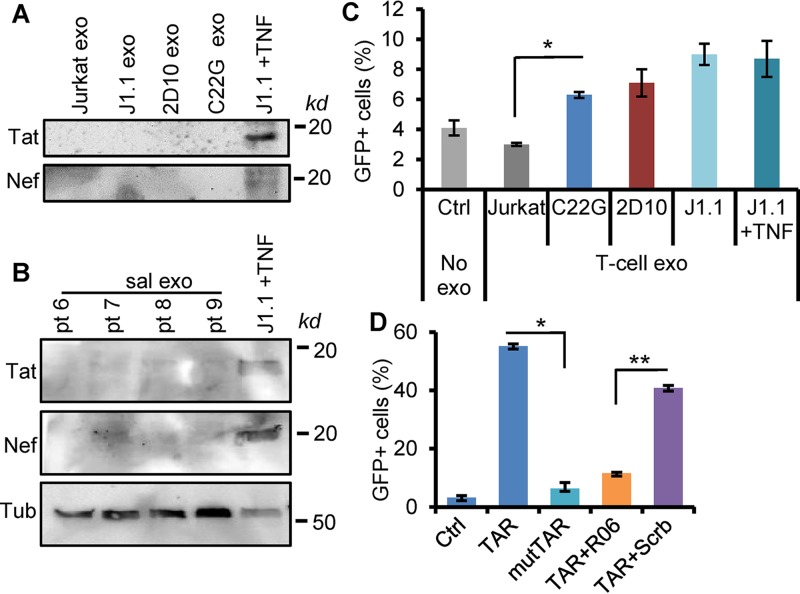
HIV TAR is critical for HIV^+^ exosome-enhanced KSHV infection. (A) Immunoblots of HIV Tat and Nef proteins on total proteins (100 μg) extracted from exosomes isolated from the culture supernatants of Jurkat, J1.1, 2D10 (26), and C22G cells that lack Nef and a functional Tat gene. Total cell lysates of J1.1 cells activated with 5 ng/ml of TNF-α (J1.1 +TNF) were used as controls. (B) Immunoblots of HIV Tat and Nef proteins on total proteins (100 μg) extracted from saliva exosomes (sal exo) purified from HIV^+^ donors (pt 6, pt 7, pt 8, and pt 9). Total cell lysates (100 μg of protein) of J1.1 cells treated with TNF-α (J1.1 +TNF) were used as controls. Tub, human tubulin, used as a loading control. (C) KSHV infection of OKF6/TERT2 cells in the presence of exosomes from Jurkat, C22G, 2D10, J1.1, and J1.1 T cells treated with TNF-α. GFP^+^ cells were determined by flow cytometry (*n* = 3). *, *P* < 0.05. (D) OKF6/TERT2 cells were transfected with the synthetic HIV TAR RNA (TAR), the mutant TAR (mutTAR), and TAR RNA together with the R06 aptamer (TAR+R06) or with the scrambled aptamer (TAR+Scrb), followed by KSHV infection for 20 h. KSHV infection was determined by GFP flow cytometry. Data represent those from one independent experiment (*n* = 4) out of three repeats. *, *P* < 0.01; **, *P* < 0.02.

We suspected that HIV-specific exosome cargo components were responsible for HIV^+^ exosome-enhanced KSHV infection in oral epithelial cells. Latently HIV-infected J1.1 T-cell exosomes do not contain HIV-1 particles, although these exosomes have viral proteins, such as Gag and the precursor form of Env protein (p160), as shown by proteomics (24). In addition, J1.1 cells can be reactivated by tumor necrosis factor alpha (TNF-α) to express viral proteins and assemble infectious virions (36). To determine if HIV^+^ exosomes contained viral proteins, such as Tat and Nef, that are known to contribute to cellular function (41, 42), we performed immunoblot assays on total exosome proteins isolated from latently HIV-infected J1.1 cells; cells of the HIV^+^ Jurkat-based C22G cell line, which contain a disruptive HIV *tat* mutant and *nef* deletion (43); and cells of the 2D10 cell line, which lack the viral *nef* gene (44). While the whole-protein lysates from TNF-α-activated J1.1 cells (26) expressed the Tat and Nef proteins, exosomes from J1.1 and C22G cells did not contain these HIV proteins (Fig. 5A). Similarly, HIV^+^ saliva exosomes did not have the Tat and Nef proteins (Fig. 5B). These results suggest that neither the Tat nor the Nef protein plays a major role in promoting KSHV infection in response to HIV^+^ exosomes. We have reported that exosomes from both the J1.1 and C22G cell lines contain HIV *trans*-activation response element (TAR) RNA, which is required for the HIV^+^ exosome-enhanced proliferation of cancer cells and extracellular signal-regulated kinase (ERK) signaling (26). HIV^+^ saliva exosomes also contained TAR RNA (Fig. 1B). Therefore, we postulated that the TAR RNA-bearing exosomes contributed to enhancing KSHV infection in oral epithelial cells. To test this hypothesis, we treated OKF6/TERT2 cells with exosomes derived from C22G, 2D10, and J1.1 cells and J1.1 cells treated with TNF-α (5 ng/ml), followed by KSHV infection. HIV^+^ exosomes from both the J1.1 and C22G cell lines increased KSHV infectivity in OKF6/TERT2 cells, as shown by GFP flow cytometry (Fig. 5C), suggesting that TAR RNA contributed to HIV^+^ exosome-enhanced KSHV infectivity. Exosomes from TNF-α-activated J1.1 cells also stimulated KSHV infection in oral epithelial cells at the same level as those from latently infected T cells, suggesting that the reactivation of HIV might not potentiate the proinfection effect of HIV^+^ exosomes. HIV TAR RNA can directly induce the expression of pro-oncogenes and the proliferation of cancer cells (26). The protumor effect of TAR RNA requires the bulge-loop structure of the molecule (45). To determine whether the bulge-loop region of TAR RNA was involved in enhancing KSHV infection, we transfected OKF6/TERT2 cells with a synthetic TAR RNA, consisting of a TAR RNA mutant containing 5 nucleotide replacements in the bulge and loop sequences (26), followed by KSHV infection assays. Our results indicate that while the wild-type TAR RNA increased KSHV infection in oral epithelial cells, the mutant TAR RNA failed to affect KSHV infection (Fig. 5D, TAR versus mutTAR). In addition, the RNA aptamer R06, which is complementary to the TAR RNA apical region and which blocks the TAR function without disrupting the secondary structure of the TAR (46), inhibited TAR RNA-enhanced KSHV infection in OKF6/TERT2 cells (Fig. 5D, TAR+R06). However, a scrambled aptamer (26) did not significantly affect TAR RNA-induced KSHV infectivity (Fig. 5D, TAR+Scrb). These results indicate that the bulge-loop region of the TAR RNA is critical for its function associated with the enhancement of KSHV infection in oral epithelial cells.

### HIV-associated exosomes promote KSHV infectivity in an EGFR-dependent fashion.

**FIG 6:**
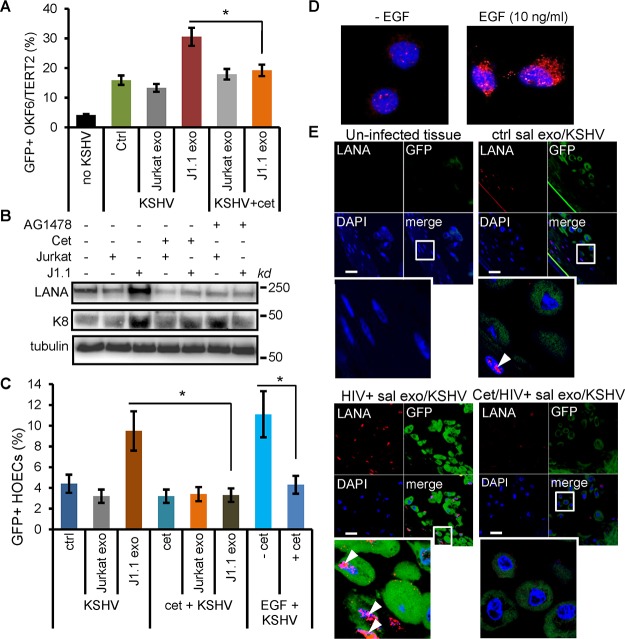
HIV^+^ exosomes enhance KSHV infection in an EGFR-dependent fashion. (A) KSHV infection in OKF6/TERT2 cells treated with exosomes from Jurkat or J1.1 cells (4 × 10^9^ exosomes/ml) with or without cetuximab (20 μg/ml). GFP^+^ cells were detected by flow cytometry. Data (mean ± SD) represent those from one independent experiment out of three repeats. no KSHV, no KSHV infection control; Ctrl, no exosome treatment control. *, *P* < 0.05. (B) OKF6/TERT2 cells were pretreated with J1.1 and Jurkat cell exosomes (4 × 10^9^ ml) in the presence or absence of cetuximab (Cet) or AG1478 (2 μm) for 30 min, followed by KSHV infection for 20 h for total protein extraction and immunoblotting of LANA and ORF K8. Tubulin was used as a loading control. (C) Flow cytometry of GFP^+^ HOECs treated with exosomes isolated from the culture supernatants of Jurkat cells or J1.1 cells or with EGF (10 ng/ml) in the presence or absence of cetuximab (20 μg/ml) (*n* = 3). *, *P* < 0.05. (D) OKF6/TERT2 cells were treated with EGF (10 ng/ml) for 10 min or remained untreated, followed by KSHV infection for 2 h. ORF65 (red) was detected by immunofluorescent staining. Representative images are shown. (E) Oral buccal mucosal tissue cultures (MatTek Inc.) were treated with J1.1 or Jurkat cell exosomes (4 × 10^9^/ml) in the presence or absence of cetuximab (20 μg/ml), followed by KSHV infection for 20 h. Cells were stained for LANA and GFP. Arrowheads, LANA; green, GFP; blue, nuclei. The lower left image represents the zoomed-in box of each merge photo to detail cellular expression of GFP and LANA.

We have reported that exosomes secreted by HIV-infected T cells and purified from the plasma of HIV-positive persons stimulate the proliferation of head and neck squamous cell carcinoma and lung cancer cells in an EGFR-dependent manner through the phosphorylation of ERK1/2 (26). We reasoned that HIV^+^ exosomes might promote KSHV infection in oral epithelial cells via the same mechanism. Indeed, the treatment of OKF6/TERT2 cells with cetuximab, a monoclonal antibody against EGFR that blocks ligand binding to the receptor (47), inhibited KSHV infection in OKF6/TERT2 cells in response to HIV^+^ exosome treatment, as determined by GFP flow cytometry (Fig. 6A). The KSHV LANA is a protein essential for the persistence of the virus (48–50) and does not exist in purified KSHV virions propagated in cultured cells (51). Open reading frame (ORF) K8 is a viral early gene encoding the ZIP family of proteins, which play a regulatory role in the immediate early and delayed-early stages of the viral life cycle (52–54). To correlate HIV^+^ exosome-enhanced KSHV infection with expression of viral proteins in host cells, OKF6/TERT2 cells were infected with KSHV virions for 20 h in response to HIV^+^ J1.1 cell exosomes in the presence or absence of EGFR inhibitors. Total protein lysates were extracted from the cells and subjected to immunoblotting for KSHV LANA and ORF K8 proteins. J1.1 cell exosomes apparently stimulated the expression of LANA in OKF6/TERT2 cells compared with Jurkat cell exosomes. The expression of the LANA protein induced by J1.1 cell exosomes was blocked by cetuximab or AG1478, an EGFR tyrosine kinase inhibitor (55) (Fig. 6B). J1.1 cell exosomes also induced ORF K8 expression, while Jurkat T-cell exosomes did not affect its expression upon KSHV infection. However, J1.1 cell exosome-induced ORF K8 expression was only partially reduced by cetuximab or AG1478 in OKF6/TERT2 cells (Fig. 6B). Since K8 is an early gene involved in the regulation of cellular pathways for full-blown viral lytic replication (53, 56, 57), these results suggest that HIV^+^ exosomes may also enhance KSHV lytic replication. To determine if cetuximab also blocked HIV^+^ exosome-enhanced KSHV infection in primary oral epithelial cells, we treated HOECs with cetuximab, followed by KSHV infection in the presence or absence of HIV^+^ J1.1 cell exosomes. HIV^+^ exosome-induced KSHV infection in HOECs was inhibited by cetuximab (Fig. 6C). To further demonstrate the involvement of EGFR in mediating KSHV infection, we also infected HOECs with KSHV in the presence or absence of recombinant EGF protein. Interestingly, EGF increased KSHV infection in HOECs, as shown by the presence of GFP^+^ cells (Fig. 6C) and *de novo* KSHV infection in OKF6/TER2 cells (Fig. 6D). Our results demonstrate the involvement of EGFR in mediating HIV^+^ exosome-enhanced KSHV infection in oral epithelial cells. To determine the effect of EGFR inhibition on KSHV infection in response to HIV^+^ saliva exosomes, we infected the oral mucosal tissue with KSHV in the presence or absence of cetuximab, followed by fluorescence microscopy for GFP and LANA. Cetuximab treatment blocked HIV^+^ saliva exosome-induced LANA expression in the oral mucosal tissue (Fig. 6E). Therefore, blocking EGFR can potentially inhibit KSHV infection mediated by HIV^+^ exosomes in the oral cavity.

### HIV-associated exosomes stimulated p38 MAPK signaling through EGFR.

**FIG 7:**
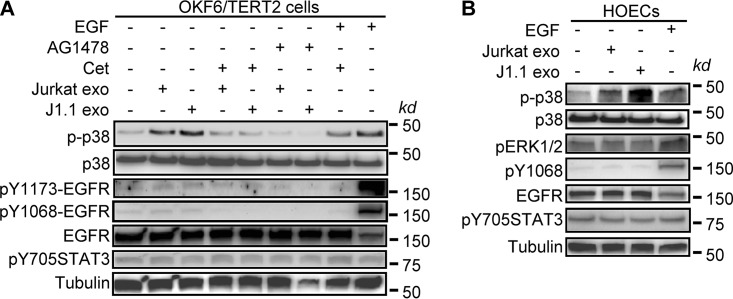
HIV^+^ exosomes activate p38 MAPK via EGFR in oral epithelial cells. (A) OKF6/TERT2 cells were pretreated with cetuximab (Cet; 20 μg/ml) or AG1478 (2 μm) for 10 min, followed by treatment with J1.1 and Jurkat cell exosomes (4 × 10^9^ exosomes/ml) for 10 min. Total protein lysates were used for immunoblotting. p-p38, phosphorylated p38; pY1173- and pY1068-EGFR, phosphorylated EGFR at tyrosine residues 1173 and 1068. EGF (10 ng/ml) was used as a positive control. (B) HOECs were treated with HIV^+^ J1.1 cell exosomes (J1.1 exo) or control Jurkat cell exosomes (Jurkat exo) (4 × 10^9^ exosomes/ml) for 10 min, followed by total protein extraction for immunoblotting. EGF (10 ng/ml) was used as a positive control.

We have reported that HIV^+^ exosomes induce the phosphorylation of ERK1/2 in an EGFR-dependent manner without causing canonical phosphorylation of the receptor in cancer cells (26). To determine if HIV^+^ exosomes contribute to the activation of EGFR and its downstream effector kinases, we treated OKF6/TERT2 cells and HOECs with exosomes isolated from HIV^+^ J1.1 and control Jurkat T cells, followed by immunoblotting of EGFR signaling mediators. Treatment of OKF6/TERT2 cells with HIV^+^ exosomes for 10 min induced the phosphorylation of p38 mitogen-activated protein kinase (MAPK), a process that was blocked by cetuximab and AG1478 (Fig. 7A). Similarly, HIV^+^ exosomes induced the phosphorylation of p38 MAPK, but not ERK1/2, in HOECs (Fig. 7B). EGF phosphorylated EGFR tyrosine residues at positions 1068 (Y1068) and 1173 (Y1173) (58–60); however, HIV^+^ exosomes failed to induce EGFR phosphorylation at these canonical sites. In addition, neither HIV^+^ exosomes nor EGF induced the phosphorylation of STAT3 at Y705 (61, 62), although these cells displayed basal levels of phosphorylation of STAT3 (Fig. 7B). Since the mitogen-activated protein (MAP) kinase pathways are well-known to control KSHV infection (63), our results suggest that TAR RNA-containing HIV^+^ exosomes interact with EGFR to activate MAPK p38, enhancing KSHV infection in oral epithelial cells.

## DISCUSSION

Saliva-mediated oral transmission of KSHV is considered the most common route for viral spreading among men who have sex with men through deep kissing and by mother-to-child transmission (3–9). HIV infection appears to play an important role in KSHV coinfection, its transmission, and its progression to malignancies (64). In people living with HIV/AIDS, coinfection with KSHV is much more likely to lead to the development of KS and other KSHV-associated diseases (65–67). In addition, the incidence rates of KSHV detection were found to be more prevalent in the HIV-infected population than in the general population in a case-control study (37). However, the mechanistic connection between HIV infection and the increased incidence of KSHV infection is unclear. In this report, we demonstrate that saliva from persons living with HIV contains HIV-associated exosomes, as shown by the presence of HIV TAR, Tat, and Nef RNAs in these extracellular vesicles. Similarly, exosomes purified from the plasma of persons living with HIV and isolated from the culture supernatants of HIV^+^ T cells contained mainly HIV TAR RNA (24, 26). Our results demonstrate that HIV^+^ exosomes enhance KSHV entry, as substantially higher numbers of virions were detected in cells treated with HIV^+^ exosomes during the early infection time. However, HIV^+^ exosomes also increased the expression of KSHV lytic protein K8. Therefore, HIV^+^ exosomes appear to not only enhance viral entry during early infection time but also increase KSHV lytic replication to promote infection.

In this report, HIV^+^ saliva samples were prepared from donors who were under ongoing ART and who had CD4 T-cell counts of >400 per ml as well as low-level viremia. HIV viral loads are correlated well in blood and semen but poorly in blood and saliva (68). It has been reported that HIV loads are generally lower in the saliva than in the blood (69). Our quantitative RT-PCR results showed that HIV-associated saliva exosomes contain HIV RNAs at levels similar to those in HIV-infected 8E5/LAV (30) T-cell exosomes. However, HIV^+^ saliva exosomes do not express HIV Env RNA, which encodes a protein forming the viral envelope (70). In addition, HIV^+^ saliva exosomes do not contain HIV Tat and Nef proteins, suggesting that our HIV^+^ saliva exosome samples did not contain HIV virions and that the proinfection effect of HIV^+^ exosomes was mainly due to TAR RNA in these exosomes. Since KSHV can be present in oral epithelial cells and is shed to cause *de novo* infection, independent of the patient’s immune status (71), and since HIV^+^ exosomes enhance *de novo* KSHV infection in oral epithelial cells, our findings suggest that HIV-associated saliva exosomes may promote KSHV transmission by increasing both the KSHV infection rate and lytic replication in oral mucosal cells.

It has been reported that oral microbial metabolites contribute to infection and the lytic activation of KSHV (33, 72, 73). Supernatants of periodontopathic bacterial cultures induce KSHV replication in cells of the BCBL-1 cell line, a KSHV latently infected lymphoma-derived cell line; embryonic kidney epithelial cells; as well as human oral epithelial cells and umbilical vein endothelial cells (72, 73). The saliva of patients with severe periodontal disease contains high levels of short-chain fatty acids that induce expression of KSHV lytic genes (73). These bacterial metabolic products can stimulate KSHV replication in infected cells using different mechanisms (72, 73). However, it is not clear whether these microbial metabolic products are responsible for KSHV infection in the oral cavity of HIV-infected persons. Collectively, our findings and these previous reports denote that multiple microbial and viral risk factors contribute to KSHV pathogenesis in the oral cavity.

Exosomes from the plasma of people living with HIV and the culture supernatants of HIV-infected T-cell lines contain HIV TAR RNA at amounts in vast excess over those of all viral mRNAs (24, 26). In patients with virtually undetectable virion levels, TAR RNA can still be found in blood exosomes (27). Our results show that HIV^+^ exosomes from saliva and T cells do not contain the HIV Tat and Nef proteins, as determined by immunoblotting. In addition, exosomes from the C22G HIV^+^ T-cell line, which contains a dysfunctional Tat mutant, which lacks the Nef gene, and which does not produce HIV virions, exhibit HIV TAR RNA and promote KSHV infection in oral epithelial cells. Therefore, our results reveal that HIV proteins and/or Tat/Nef RNA is not involved in the proinfection effect of HIV^+^ exosomes. Several reports have shown that HIV TAR RNA is a functional component of the HIV^+^ exosome cargo and induces the expression of proinflammatory cytokines and proto-oncogenes in primary human macrophages and cancer cells, respectively (24, 26, 27). Synthetic TAR RNA alone can stimulate the proliferation and migration of head and neck cancer cells (26). The mutant TAR RNA with 5 nucleotide substitutions in the bulge and loop sequences fails to induce gene expression in cancer cells (26). Similarly, our results demonstrate that, while the wild-type synthetic TAR RNA stimulates KSHV infection, the TAR RNA mutant cannot enhance KSHV infection in oral epithelial cells. In addition, the R06 nucleotide aptamer, which creates an imperfect hairpin to complement the entire TAR loop and which blocks TAR RNA function (46), attenuates TAR RNA-induced KSHV infection. The R06 aptamer and its derivatives are able to reduce HIV-1 infection and inhibit viral transcription (74, 75). Kolb et al. have reported that the replication of HIV-1 and that the activity of β-galactosidase under the control of the HIV-1 5′ long terminal repeat were reduced in cells expressing the nuclear R06 transcript (74), suggesting antiviral activity for the nucleotide aptamer. Our results suggest that the R06 RNA aptamer and its functional derivatives can potentially be developed as treatments for controlling coinfection with herpesviruses in the HIV-infected population.

We have reported that HIV^+^ exosomes stimulate the proliferation and proto-oncogene expression of squamous cell carcinoma cells in an EGFR-dependent manner (26). Similarly, EGFR is critical for HIV^+^ exosome-enhanced KSHV infectivity; blocking the receptor with cetuximab or the receptor kinase inhibitor effectively inhibits KSHV infection. Concordant with the role of EGRF in mediating KSHV infection, EGF alone increases KSHV infectivity in oral epithelial cells, a process that can be inhibited by cetuximab. In head and neck as well as lung cancer cells, EGFR mediates HIV^+^ exosome entry into target cells and participates in exosome-induced cellular signaling, including ERK1/2 phosphorylation, without activating the receptor (26). However, B-cell lymphoma cells, which lack EGFR, do not respond to the protumor effect of HIV^+^ exosomes (26). In this report, we demonstrate that HIV^+^ T-cell exosomes specifically activate MAPK p38, but not ERK1/2, through EGFR without inducing canonical receptor phosphorylation. The ERK1/2 pathway is primarily driven by EGFR *in vitro* and *in vivo* in various types of cancers and is a potential target in cancer therapy (76, 77). EGFR mediates the entry of HIV^+^ exosomes into cancer cells, possibly enriched in endosomes (26). HIV^+^ exosome-induced activation of the EGFR-ERK axis requires Toll-like receptor 3 (TLR3), an endosomal receptor that has been shown to bind to EGFR and the kinase Src residing in endosomes for TLR3 activation in response to double-stranded RNA (dsRNA) (78). Interestingly, EGF alone can promote KSHV infection in a fashion similar to that for HIV^+^ exosomes. In addition, EGF induces the phosphorylation of p38 and ERK1/2 MAPKs, suggesting that the EGFR signaling is critical for enhanced KSHV infection in oral epithelial cells. The p38 signaling may play a key role in KSHV virology. Inhibition of the catalytic activity of phosphorylated p38 blocks KSHV reactivation, possibly through a reduction in global H3 acetylation and phosphorylation (79). The MAP kinase pathways are well-known to control KSHV infection (63). In addition, several host and environmental factors have been considered to play roles in KSHV infection and reactivation. Various chromatin-silencing mechanisms, including histone deacetylation, repressive histone methylation, and DNA methylation, lead to silencing of the genomes of herpesviruses and HIV during latency. Inhibition of class 1/2 histone deacetylases (HDACs) by multiple short-chain fatty acids results in the expression of genes associated with the fate of KSHV infection and viral reactivation (81–83). The epigenetic modifications, particularly the acetylation of histones, are required for the maintenance of KSHV latency in classic and AIDS-associated KS tissues (82). Taken together, our findings provide insight into the mechanisms underlying HIV-specific components and coinfection with KSHV through the oral cavity in people living with HIV/AIDS. In addition, targeting HIV TAR RNA and EGFR of oral epithelial cells may serve as novel approaches to control KSHV infection in the HIV-infected population.

## MATERIALS AND METHODS

### Ethical statement.

For all human subject studies, written informed consent was obtained from all study participants, according to a protocol approved by the Human Subjects Institutional Review Board (IRB) at Case Western Reserve University and University Hospitals Cleveland Medical Center. Only deidentified human specimens were collected and used for this work.

### Cells, cell cultures, and reagents.

The J1.1 and 8E5/LAV cell lines were obtained from the NIH AIDS Reagent Program. C22G and 2D10 cells were gifts from J. Karn (Case Western Reserve University). Jurkat cells were purchased from the American Type Culture Collection (TIB-152; ATCC, Manassas, VA). These cells were maintained in RPMI 1640 medium (HyClone Laboratories, Inc., Logan, UT) supplemented with 10% exosome-depleted fetal bovine serum (FBS), which was prepared by ultracentrifugation of FBS (Thermo Fisher Scientific, Waltham, MA) at 100,000 × *g* for 16 h at 4°C (26), followed by collection of the supernatants without disturbing the pellet. Primary human oral epithelial cells (HOECs) were isolated from healthy donors who underwent third-molar extraction at the Case Western Reserve University School of Dental Medicine as previously described (84). OKF6/TERT2 immortalized human keratinocytes were obtained from J. G. Rheinwald (34). HOECs and immortalized OKF6/TERT2 cells were maintained in keratinocyte-serum-free medium (KSFM; catalog number 17005-042; Invitrogen) as previously described (34, 85). EpiOral oral mucosal tissues were purchased from MatTek Co. (Ashland, MA). These tissues consist of normal human oral keratinocytes that are differentiated into tissues with a noncornified, buccal phenotype. The 3-D organotypic cultures were constructed following protocols previously published by Dongari-Bagtzoglou and Kashleva (39). Briefly, a collagen gel cushion was prepared on ice from rat tail type I collagen (catalog number Corning 354249; Thermo Fisher Scientific) supplemented with 10% FBS in Dulbecco modified Eagle medium (DMEM) and antibiotics. The fibroblast gel layer was prepared by mixing 1 ml of NIH 3T3 cells with the collagen gel, as mentioned above. Culture inserts containing the gel cushion and the fibroblast gel layer were cultured for 4 days, followed by addition of OKF6/TERT6 cells to the center of the insert and culture for 3 days. These inserts were then lifted and cultured in airlifting medium for 14 days, with a change of the medium every other day.

Primer sequences for PCR amplimers were as follows: for TAR RNA (60 bp), forward primer 5′-GGTCTCTCTGGTTAGACC and reverse primer 5′-GTGGGTTCCCTAGTTAGC; for Tat (192 bp), forward primer 5′-GAAGCATCCAGGAAGTCAGC and reverse primer 5′-GGAGGTGGGTTGCTTTGATA; for Nef (175 bp), forward primer 5′-ATTGGATGGCCTGCTGTAAG and reverse primer 5′-GGAAAACCCACCTCTTCCTC; and for Env (168 bp), forward primer 5′-GGCAAGTCTGTGGAATTGG and reverse primer 5′-TGGGATAAGGGTCTGAAACG.

The antibodies used in immunoblotting and immunofluorescence microscopy were CD9 (clone TS9, catalog number 10626D), CD81 (clone M38, catalog number MA1-10290), and rabbit polyclonal claudin 1 (catalog number 51-9000) antibodies from Thermo Fisher Scientific; CD63 (clone NKI/C3, catalog number NBP2-32819) and KSHV K8 (clone 8C12G10G1, catalog number NB-100-1086) antibodies from Novus Biologicals; pY1175-EGFR (catalog number 05-483), EGFR (catalog number 4267), phopho-p38 (catalog number 9211), p38 (clone D13E1, catalog number 8690), and phospho-p38 (catalog number 9211) antibodies from Cell Signaling Technology; KSHV LANA antibody (clone LN53, catalog number MABE1109) from Millipore; and GFP antibody (catalog number 13970) from Abcam. The mouse monoclonal antibody to KSHV ORF65 was a kind gift from Shoujian Gao at the University of Texas Health Science Center, San Antonio, TX.

### Preparation of KSHV virions and exosomes.

KSHV was prepared as described by Brulois et al. (31). Briefly, iSLK-BAC16 cells were maintained in DMEM supplemented with 10% FBS, antibiotics (antibiotic-antimycotic; catalog number 15240-062; Life Technologies), Primocin (catalog number ANTPM1; InvivoGen), and hygromycin (catalog number 89150-426; VWR). iSLK-BAC16 cells (∼70% confluent) were then activated by 1 mM sodium butyrate (catalog number 303410-100G; Sigma-Aldrich, ) and 1 μg/ml of doxycycline (catalog number DSD43020-10; Dot Scientific, Burton, MI) for 2 days, and then fresh medium (DMEM with 10% FBS only) was added to the cells for 3 days. The supernatants were collected and centrifuged to remove the cell debris. The resulting supernatants were centrifuged at 10,000 × *g* for 10 min, and the KSHV pellet was suspended with base KSFM. The titer of the virus was determined by infection of OKF6/TERT2 cells with serial dilutions of KSHV. Exosomes were prepared from the cell supernatants by differential ultracentrifugation with filtration steps (26). Briefly, cell culture medium or saliva samples were centrifuged at 400 × *g* for 5 min to remove the cells, followed by centrifugation at 11,000 × *g* for 10 min to remove any possible apoptotic bodies and large cell debris. The exosomes were then precipitated at 100,000 × *g* for 90 min at 4°C and suspended in phosphate-buffered saline (PBS). Isolated exosomes were quantified using the acetylcholinesterase (AChE) assay system (26) (System Biosciences Inc./SBI, Palo Alto, CA) (for exosomes from the T-cell lines) or total protein concentrations (for exosomes from saliva).

### Cryo-EM imaging.

Exosomes purified from the culture supernatants of T cells and saliva were used for cryo-EM imaging. To reduce the viscosity of the saliva sample, a brief 10-s sonication was performed after ultracentrifugation. Quantifoil grids (2/2, 200 mesh) were glow discharged using an EMITech K100X glow discharge unit for 60 s at 30 mA. Small aliquots (3 μl) from each of the purified samples were applied to the Quantifoil grids, blotted and cryo-plunged with an FEI Vitrobot. Cryo-electron micrographs were acquired with an FEI Tecnai TF20 (200 kV; field emission gun) cryo-transmission electron microscope operating at an accelerating voltage of 200 kV. Images were collected on a Tietz video and image processing system (TVIPS) TemCam-F416 (4k × 4k) complementary metal oxide semiconductor camera at a nominal magnification of ×25,500 and with a defocus ranging from −3 μm to −5 μm.

### Flow cytometry analysis.

OKF6/TERT or HOECs were washed 3 times with PBS and then suspended in 100 μl of PBS. Flow cytometric analysis was performed by green fluorescent protein (GFP) and/or LANA staining on a FACSAria flow cytometer (BD Biosciences). Fluorescence-activated cell sorting data were analyzed with FlowJo software (TreeStar Inc.).

### RT-PCR, immunoblotting, and immunofluorescence microscopy.

Total RNA was isolated and purified from exosomes using a High Pure RNA isolation kit (Roche) according to the manufacturer’s instructions. Extracted RNA was reverse transcribed to cDNA (High-Capacity cDNA reverse transcription; Applied Biosystems). PCR was performed with the Q5 Hot Start high-fidelity 2× master mix (New England Biolabs) on the T100 thermal cycler (Bio-Rad) using the primers described previously (26).

For immunoblotting, total exosome proteins were purified using a Total Exosome RNA & Protein isolation kit (Thermo Fisher Scientific) following the manufacturer’s instructions. To prepare total cellular proteins, cells were washed with PBS, and then the cellular lysates were obtained by adding 300 μl of radioimmunoprecipitation assay lysis and extraction buffer (Thermo Fisher Scientific). Protein lysates were separated by SDS-PAGE and then transferred onto polyvinylidene fluoride (PVDF) membranes (Merck Millipore) for immunoblot analysis. Protein detection was performed by chemiluminescence using an enhance chemiluminescence kit (Thermo Fisher Scientific) with a ChemiDoc XRS+ imaging system (Bio-Rad).

Immunofluorescence microscopy was performed as previously described (86), with minor modifications. Briefly, each section (5 μm) of organotypic cultures or the oral mucosal tissues was deparaffinized 3 times in Clear-Rite 3 reagent (Thermo Fisher Scientific), hydrated with 100% alcohol followed by 95% alcohol, and washed with PBS. Samples were blocked with 10% donkey serum at room temperature for 1 h. Each section was incubated with the primary antibody at 4°C overnight. After washing in PBS, sections were stained with Alexa Fluor-conjugated secondary antibody appropriate to the species of the primary antibody. Sections were then mounted with Vectashield fluorescent mounting medium (Vector Laboratories Inc., Burlingame, CA) containing DAPI (4′,6-diamidino-2-phenylindole) to visualize the nuclei. Immunofluorescent images were generated using an AMG Evos FL digital inverted fluorescence microscope (AMG, Mill Creek, WA). Confocal images were acquired with a Leica TCS SP8 system (Leica Microsystems) using a 63× (numerical aperture, 1.4) objective at a pixel size of 90 nm. For fluorescence immunocytochemistry, cells on an 8-well culture slide were fixed with 100% methanol at −20°C for 20 min, followed by permeabilization with 0.3% Triton X-100 in PBS. The cells were then stained with the primary antibody, followed by incubation with appropriate secondary antibodies. Fluorescent images were taken as described above. Micrographic images were processed using ImageJ software.

### Statistics.

The results obtained with the treatments were compared with those obtained for the respective controls. Data are represented as the mean ± standard deviation (SD). Flow cytometry data were subjected to one-way analysis of variance (ANOVA). Statistical significance was considered at *P* values of <0.05. For donors *n* ≤ 5, the F test was applied. Data analyses were performed and graphs were generated using Prism (GraphPad Software, La Jolla, CA) and Excel 2013 (Microsoft Inc. Redmond, WA) software.
